# Relationship between uric acid and depression in American adults: findings from NHANES, 2005-2020

**DOI:** 10.3389/fpsyt.2025.1544266

**Published:** 2025-06-20

**Authors:** Pengwei Lai, Xingyun Xie, Wen Zeng, Weiwen Cheng, Xinyuan Liu, He Long, Taierqi Peng, Antong Hu, Xiaohong Du

**Affiliations:** ^1^ The Second Affiliated Hospital, Jiangxi Medical College, Nanchang University, Nanchang, Jiangxi, China; ^2^ Department of Anesthesiology, The Second Affiliated Hospital, Jiangxi Medical College, Nanchang University, Nanchang, Jiangxi, China

**Keywords:** uric acid, depression, NHANES, disease marker, public health

## Abstract

**Background:**

At present, the role of uric acid in mental disorders is receiving increasing attention, but its relationship with depression is controversial, and previous studies have corresponding limitations. The relationship between them has not been fully elucidated.

**Objective:**

The purpose of this study is to explore the relationship between uric acid and depression in American adults using data from the National Health and Nutrition Examination Survey (NHANES).

**Methods:**

This cross-sectional study included participants from the National Health and Nutrition Examination Survey 2011-2020. Use logistic regression and restricted cubic spline analysis to investigate the relationship between uric acid and depression. The interactions between variables were determined using subgroup analysis and described in a forest plot.

**Results:**

37033 participants were included in this study, with depression patients accounting for 8.95%. The uric acid levels in the depression group and nondepression group were 317.69 μ mol/L and 323.34 μ mol/L, respectively, with the former having significantly lower uric acid levels than the latter. In the fully adjusted model, participants in the third tertile of uric acid showed a significant correlation (P=0.002, OR; 0.85 (0.76 ~ 0.94)) with a higher risk of depression compared to participants in the first tertile. There is an approximately linear negative correlation between uric acid and depression (P for nonlinear=0.360), and the OR value of uric acid is 1 when the uric acid value is 312.20 μ mol/L.

**Conclusions:**

Current research suggests that serum uric acid is associated with depression in American adults. More discoveries and causal relationships require further investigation.

## Graphical Abstract

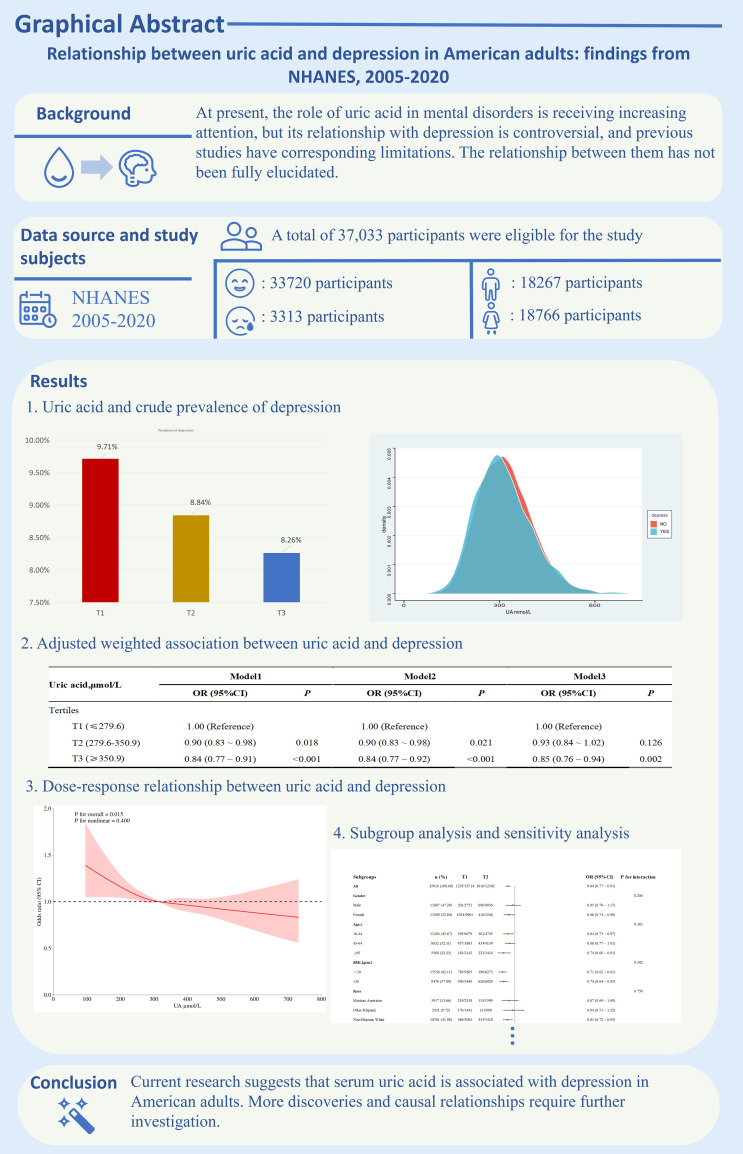

## Introduction

As people’s awareness of mental illnesses continues to deepen, depression has garnered increasing attention (1). Depression is defined as a prolonged period of low mood or a loss of pleasure or interest in activities (2). It is estimated that approximately 5% of adults worldwide suffer from depression each year, and this figure is still on the rise (1, 3). The diagnosis and treatment of depression are also not optimistic. In developed countries, about half of depressed patients do not receive proper diagnosis and treatment, while in developing countries, this figure increases to 80%-90% (4). Patients with depression who do not receive proper diagnosis and treatment can appear in various populations, causing immeasurable losses to individuals, families, and society (3, 5, 6). Studies have shown that major depression will become the biggest burden of non-fatal health loss by 2030 (3, 7).

Uric acid is a well-known substance, which is the ultimate product of the metabolism of adenine and guanine (8). In the past, uric acid was often regarded as a metabolic waste, but now people are paying more and more attention to its physiological role. The role of uric acid is diverse. It can react with various oxidants to form free radicals (9), reduce the bioavailability of NO, inhibit cell migration and endothelial cell proliferation, and promote oxidation and inflammation (10–14), It can also scavenge superoxide anions and inhibit the formation of nitrated tyrosine (15, 16), thus achieving anti-inflammatory and antioxidant protective effects (17–20). In addition, it can also affect changes in white matter connectivity (21). The interaction of various mechanisms is related to uric acid concentration, the type and concentration of free radicals, the patient’s own state, and the type of disease, presenting a very complex state (22, 23).

There is much debate about the relationship between uric acid and depression, with varying effects observed in different regions and populations. A study in the elderly population in China found that the prevalence of depression among uric acid-hypertension participants was not high in women, with a negative correlation between uric acid and depression, which was not found in men (24). However, another cross-sectional study showed that higher serum uric acid was associated with depression in postmenopausal women (25). In the elderly population in South Korea, uric acid was negatively correlated with depression in elderly women but was unrelated to depression in elderly men (26). A study on depression and anxiety from the Netherlands showed that patients with severe depression had lower uric acid levels, but there were no confounding factors such as diet (27). However, the results of a Mendelian randomization study from Europe and South America suggest that there is no significant causal relationship between serum uric acid and severe depression from a genetic perspective (28).

The relationship between uric acid and depression has been reported inconsistently in the literature, with some studies even yielding contradictory findings. Certain studies have been limited by the absence of relevant confounding factors, inadequate sample sizes, or restricted conclusions (24–28). Consequently, it is crucial to address these existing limitations. Thus, this study aims to investigate the association between uric acid levels and depression among adults in the United States utilizing data from the National Health and Nutrition Examination Survey (NHANES).

## Methods

### Data source

NHANES is a continuous, nationally representative survey that will be used to assess the nutritional status and its association with health promotion and disease prevention (29). This study was exempt from institutional review board review because the NHANES dataset is unidentified and publicly available.

### Variable definitions

The Patient Health Questionnaire-9 (PHQ-9), first published in 2001, has been included in numerous depression guidelines and implemented in many clinical practice settings, becoming a widely recognized questionnaire for depression assessment (30). NHANES began using the PHQ-9 as a depression diagnostic criterion in 2005. Therefore, we selected data from survey participants from 2005 to the latest (i.e., 2005-2020) as the research object.

We consider a PHQ-9 score of 10 or higher as a diagnosis of depression. The uric acid data is from the Standard Biochemistry Profile in Laboratory Data. Other variables included are: gender (male and female); age (years) (18-44, 45-64,and≥65); race/ethnicity (Mexican American, Other Hispanic, Non-Hispanic Black, and Other Race); educational level (Less than 9th grade, 9-11th grade, High school graduate, Some college or AA degree,and College graduate or above); Marital status (Married/Living with Partner, Widowed/Divorced/Separated and Never married); Family monthly poverty level category (≤1.30, 1.30 -1.85, >1.85, Refused and Don’t know); Moderate work activity-defined as at least 10 minutes of moderate activity that increases the participant’s breathing and heart rate (yes or no); Sleeping trouble-defined as having ever told a doctor or other health professional that you have trouble sleeping (yes or no); Smoking: defined as having smoked at least 100 cigarettes in a lifetime (yes or no); Drinking-defined as having consumed at least 12 alcoholic beverages in one’s lifetime (yes or no); High blood pressure(yes or no); High cholesterol level(yes or no); Diabetes (yes or no); BMI (kg/m2) (<30, ≥30); Total energy intake (kcal/day) (≤2100, >2100) and serum creatinine levels(μmol/L)(≤44, 44 -133, >133). We also collected information on the use of medications for hypertension, hyperlipidemia, and diabetes whenever possible. Additionally, we gathered detailed data on the types and quantities of foods and beverages (including all types of water) consumed in the 24 hours preceding the survey (from midnight to midnight) and converted this information into various dietary nutrients.

### Statistical analysis

Continuous variables with normally distributed data are expressed as mean ± standard deviation (SD), while continuous variables with non-normally distributed data are expressed as median within the range of tertiles, and categorical data are displayed as frequency percentages. The baseline characteristics of depressive symptoms were evaluated using independent two-sample t-tests for continuous variables and chi-square tests for categorical variables. Binary logistic regression analysis was used to determine the odds ratio (OR) and 95% confidence interval (CI) between uric acid and depression. The relationship between uric acid concentration and depression was evaluated through the RCS curve. Use subgroup analysis to test interactions and display adjusted OR and 95% CI in forest plots. Compare the third tertile and the first tertile of uric acid in subgroup analysis to enhance statistical power. In all analyses, a p-value of < 0.05 on both sides was considered statistically significant. All data analysis was performed using R software version 4.1.3 (www.R-project.org) and SPSS software (version 26; IBM Corp; Armonk, NY, USA).

## Results

### Characteristics and parameters of the participants

**Figure 1:**
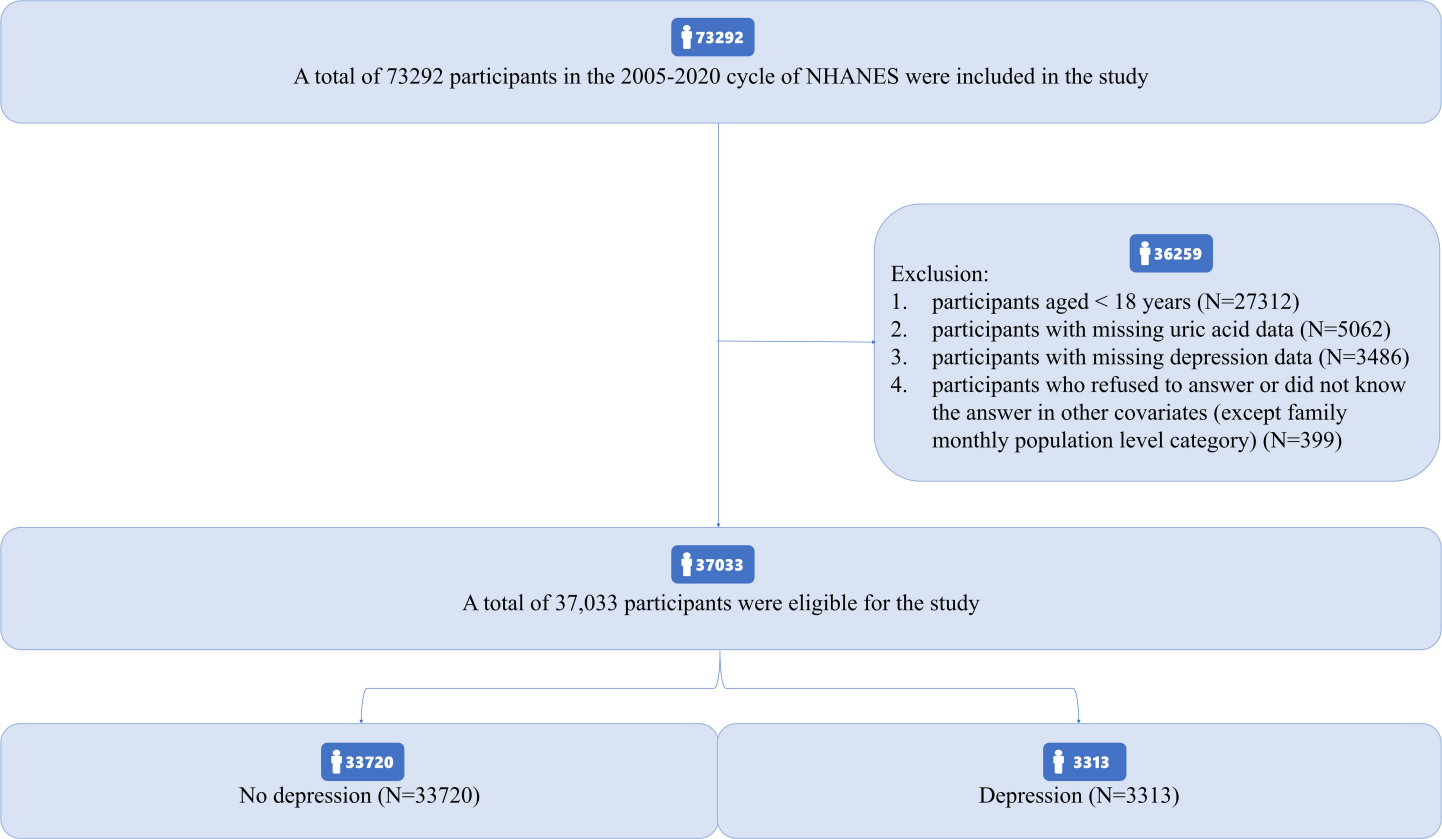
Study flow diagram.

As shown in **Figure 1**. A total of 73292 participants were included in the NHANES cycle from 2005 to 2020, including 27312 participants aged < 18 years, 5062 participants with missing serum uric acid data, 3486 participants with missing depression data, 399 participants who refused to answer or did not know in other included variables (except family monthly poverty level category), and only 37033 participants were finally included in this study. There were 33720 participants without depression and 3313 patients with depression, accounting for 8.95%. The prevalence was consistent with that of the general population in the United States in other studies (31).

**Table 1** shows that the uric acid levels of the depression group and non-depression group are 317.69 μ mol/l and 323.34 μ mol/l respectively, and the serum uric acid level of the former is significantly lower than that of the latter. In addition, compared with the non-depression group, the BMI of the depression group was higher, but the total energy intake was lower. Men and women accounted for almost half of the total population respectively, but the proportion of women in the depression group was significantly greater than that of men, accounting for 62.66%. In addition, within the race, education level, marital status, and family monthly poverty level category groups, there were statistically significant differences between the depression group and the non-depression group (P <0.001). Finally, participants without moderate work activity and with sleeping trouble, smoking, drinking, high blood pressure, high cholesterol level, and diabetes accounted for a higher proportion in the depression group (P <0.001). The specific details regarding the intake of various dietary nutrients and medications are provided in **Supplementary Table 1**. Compared with individuals in the general population, those with depression consumed less protein, fiber, and various antioxidant-rich foods but more sugar and caffeine. There were no significant differences between the two groups in the use of antihypertensive and lipid-lowering medications, while the use of diabetes medications was more prevalent among individuals with depression.

**Table 1:** Characteristics of the study population.

Characteristics	Total (n = 37033)	depression (n = 33720)	Normal (n = 3313)	*P*
Uric acid,μmol/L	322.84 ± 85.92	323.34 ± 85.64	317.69 ± 88.57	<0.001
Age,y	47.90 ± 18.62	47.88 ± 18.75	48.06 ± 17.33	0.574
BMI, kg/m2	29.18 ± 7.06	29.01 ± 6.89	30.88 ± 8.40	<0.001
Total energy intake, kcal/day,	2124.37 ± 1005.49	2133.13 ± 1000.52	2035.19 ± 1050.82	<0.001
Serum creatinine,μmol/L	79.81 ± 40.12	79.80 ± 38.07	79.99 ± 56.94	0.844
Gender, n(%)				<0.001
Male	18267 (49.33)	17030 (50.50)	1237 (37.34)	
Female	18766 (50.67)	16690 (49.50)	2076 (62.66)	
Race, n(%)				<0.001
Mexican American	5876 (15.87)	5360 (15.90)	516 (15.58)	
Other Hispanic	3598 (9.72)	3173 (9.41)	425 (12.83)	
Non-Hispanic White	15591 (42.10)	14232 (42.21)	1359 (41.02)	
Non-Hispanic Black	8022 (21.66)	7278 (21.58)	744 (22.46)	
Other Race	3946 (10.66)	3677 (10.90)	269 (8.12)	
Education level, n(%)				<0.001
Less than 9th grade	3494 (9.43)	3030 (8.99)	464 (14.01)	
9-11th grade	5120 (13.83)	4460 (13.23)	660 (19.92)	
High school graduate	8542 (23.07)	7731 (22.93)	811 (24.48)	
Some college or AA degree	11267 (30.42)	10260 (30.43)	1007 (30.40)	
College graduate or above	8610 (23.25)	8239 (24.43)	371 (11.20)	
Marital status, n(%)				<0.001
Married/Living with Partner	21631 (58.41)	20119 (59.66)	1512 (45.64)	
Widowed/Divorced/Separated	7750 (20.93)	6690 (19.84)	1060 (32.00)	
Never married	7652 (20.66)	6911 (20.50)	741 (22.37)	
Family monthly poverty level category, n(%)				<0.001
≤1.30	12506 (33.77)	10773 (31.95)	1733 (52.31)	
1.30 -1.85	5219 (14.09)	4721 (14.00)	498 (15.03)	
>1.85	18472 (49.88)	17467 (51.80)	1005 (30.34)	
Refused	220 (0.59)	208 (0.62)	12 (0.36)	
Don't know	616 (1.66)	551 (1.63)	65 (1.96)	
Moderate work activity, n(%)				<0.001
Yes	15339 (41.42)	14100 (41.81)	1239 (37.40)	
No	21694 (58.58)	19620 (58.19)	2074 (62.60)	
Sleeping trouble, n(%)				<0.001
Yes	9354 (25.26)	7477 (22.17)	1877 (56.66)	
No	27679 (74.74)	26243 (77.83)	1436 (43.34)	
Smoking, n(%)				<0.001
Yes	16166 (43.65)	14268 (42.31)	1898 (57.29)	
No	20867 (56.35)	19452 (57.69)	1415 (42.71)	
Drinking, n(%)				<0.001
Yes	27090 (73.15)	24577 (72.89)	2513 (75.85)	
No	9943 (26.85)	9143 (27.11)	800 (24.15)	
High blood pressure, n(%)				<0.001
Yes	12744 (34.41)	11225 (33.29)	1519 (45.85)	
No	24289 (65.59)	22495 (66.71)	1794 (54.15)	
High cholesterol level, n(%)				<0.001
Yes	13367 (36.09)	11933 (35.39)	1434 (43.28)	
No	23666 (63.91)	21787 (64.61)	1879 (56.72)	
Diabetes, n(%)				<0.001
Yes	4548 (12.28)	3928 (11.65)	620 (18.71)	
No	31652 (85.47)	29059 (86.18)	2593 (78.27)	

Continuous variables are expressed as mean (SD) for normally distributed data and as median with interquartile range for non normally distributed data. Categorical variables are expressed as percentages.

### Relationship between uric acid and risk of depression

As shown in **Table 2**, we evaluated the risk relationship between uric acid and depression in the original model and the adjusted model respectively. In the original model, compared with participants in uric acid tertile 1, participants in uric acid tertiles 2 and 3 were significantly associated with the risk of depression, with P and OR values of P=0.018, OR:0.90 (0.83 ~ 0.98), P<0.001, OR:0.84 (0.77 ~ 0.91), respectively. Model 2 included age and gender in the analysis, and the final results were consistent with the original model. After additional adjustments for gender, age, race/ethics, educational level, marital status, family monthly population level category, moderate work activity, sleeping trouble, smoking, drinking, high blood pressure, high cholesterol level, diabetes, BMI, total energy intake and serum creatinine levels, model 3 still found that participants with serum uric acid tertile 3 had significantly higher risk of depression than those with tertile 1 (P=0.002, OR:0.85 (0.76 ~ 0.94)).In **Supplementary Table 2**, we added dietary nutrients and medication use as confounding factors and adjusted for them. The results remained robust (P = 0.033, OR: 0.81 (0.66–0.98)).

**Table 2:** The relationship between uric acid and the risk of depression.

Uric acid,μmol/L	Model1		Model2		Model3	
	OR (95%CI)	*P*	OR (95%CI)	*P*	OR (95%CI)	*P*
Tertiles						
T1 (≤279.6)	1.00 (Reference)		1.00 (Reference)		1.00 (Reference)	
T2 (279.6-350.9)	0.90 (0.83 ~ 0.98)	0.018	0.90 (0.83 ~ 0.98)	0.021	0.93 (0.84 ~ 1.02)	0.126
T3 (≥350.9)	0.84 (0.77 ~ 0.91)	<0.001	0.84 (0.77 ~ 0.92)	<0.001	0.85 (0.76 ~ 0.94)	0.002

OR, Odds Ratio; CI, Confidence Interval; Model1: Crude; Model2: Adjust: Age, Race; Model3: Adjust: Age, Gender, BMI, Race, Education level, Marital status, Family monthly poverty level category, Moderate work activity, Sleeping trouble, Smoking, Drinking, Total energy intake, High blood pressure, High cholesterol level, Diabetes, Serum creatinine.

### Dose-response relationship between uric acid and depression

**Figure 2:**
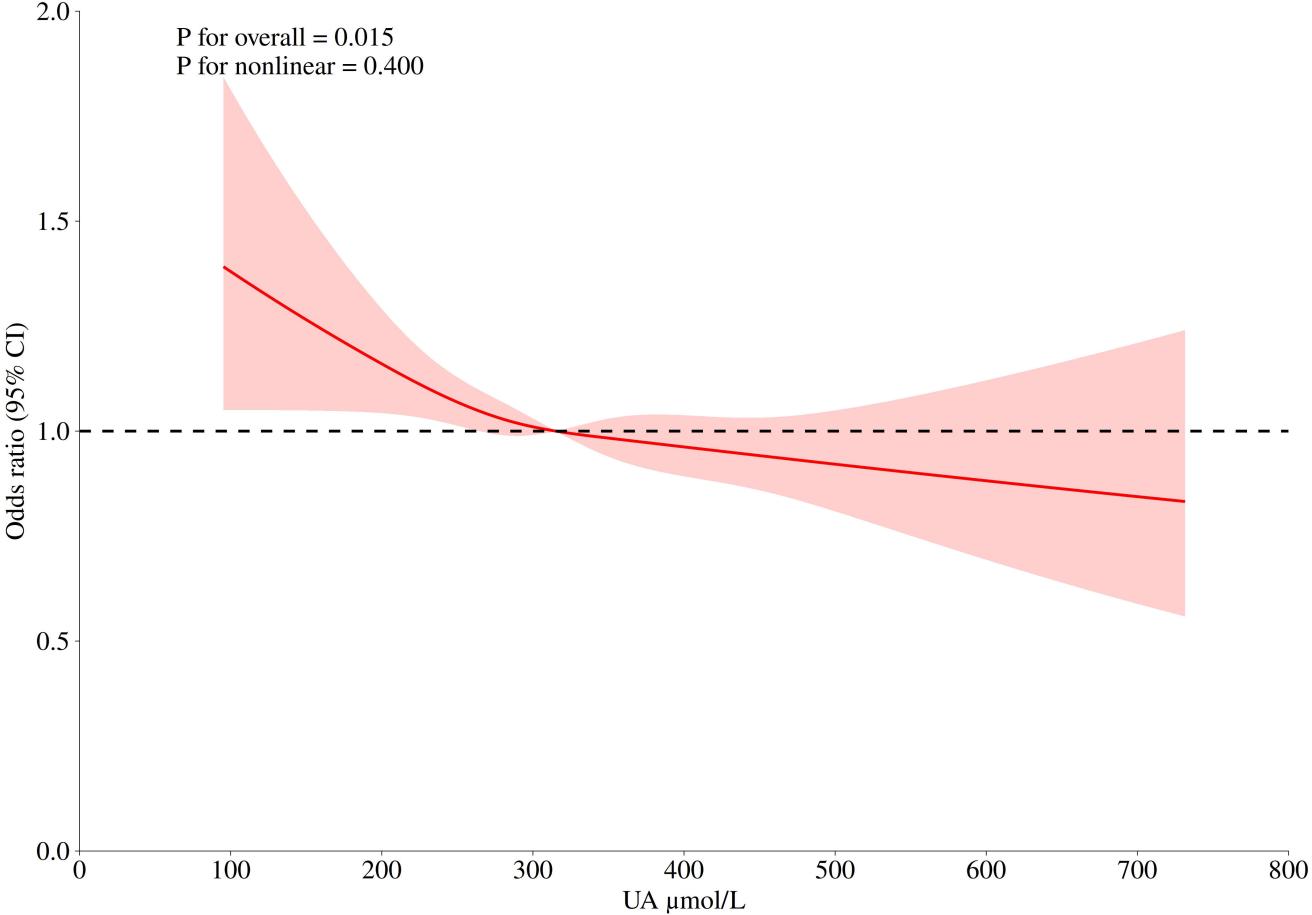
The logistic risk regression model of uric acid and depression, the adjusted hazard ratio (95% CI) of uric acid and depression is represented by a red curve. The cubic spline curve of the model has four nodes. Adjusted confounders included: Age, Gender, BMI, Race, Education level, Marital status, Family monthly poverty level category, Moderate work activity, Sleeping trouble, Smoking, Drinking, Total energy intake, High blood pressure, High cholesterol level, Diabetes, Serum creatinine.

**Figure 2** shows the dose-response relationship between uric acid concentration and depression risk. It can be seen from the figure that the curve shows a downward trend. There is an approximately linear negative correlation between uric acid and depression (P for nonlinear=0.400), and the optimal cutoff value is 315.20 μ mol/L. It means that when the serum uric acid concentration is less than this value, the OR value is greater than 1, and the risk of depression is significantly increased. Similarly, after the uric acid concentration exceeds 315.20 μ mol/L, uric acid is a protective factor for the occurrence of depression, and on the whole, the uric acid concentration is statistically significant for the occurrence of depression (P for overall = 0.015). In addition, in order to avoid the interference caused by dietary intake and common drugs, we adjusted their effects in **Supplementary Figure 1**, and the restricted cubic spline (RCS) curve still showed a similar trend.

### Subgroup analysis and sensitivity analysis

**Figure 3:**
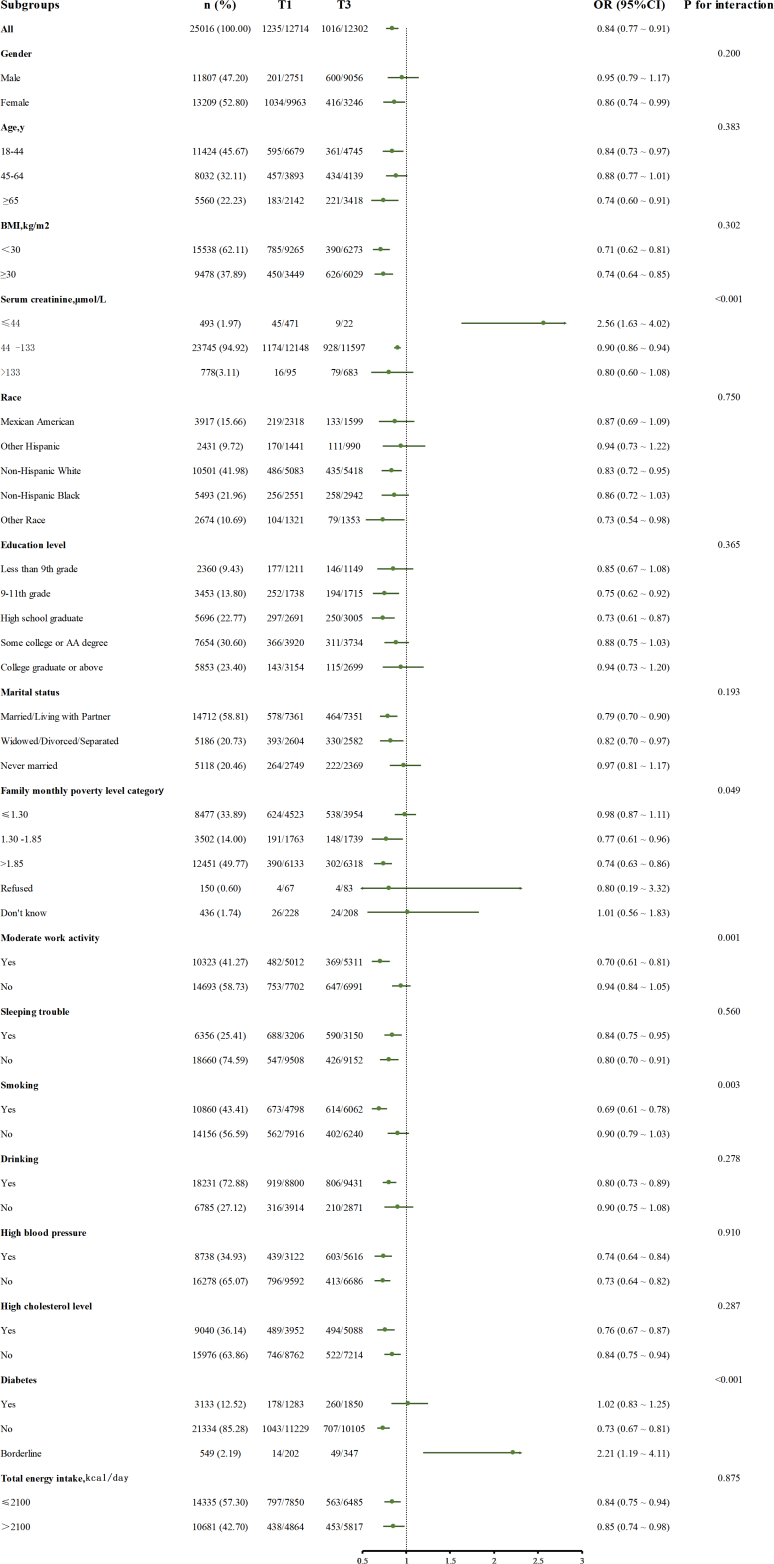
The relationship between serum uric acid (T3 and T1) and depression risk in different subgroups, adjusted for confounding factors included: Age, Gender, BMI, Race, Education level, Marital status, Family monthly poverty level category, Moderate work activity, Sleeping trouble, Smoking, Drinking, Total energy intake, High blood pressure, High cholesterol level, Diabetes, Serum creatinine.

In **Figure 3**, the subgroup analysis of serum uric acid concentration (T3 vs T1) and depression risk showed that in 25016 cases of the total population, high uric acid level (T3 group) was significantly associated with reduced depression risk (OR=0.84, 95%CI: 0.77 – 0.91). Subgroup analysis found that the association was affected by serum creatinine level and lifestyle factors: in the population with normal serum creatinine, the risk of depression in T3 group was lower (OR=0.90, 95%CI: 0.86 – 0.94), but there was a risk reversal in the group with creatinine ≤ 44 μ mol/L (OR=2.56, 95%CI: 1.63 – 4.62, P for interaction <0.001), and the risk relationship was consistent with the overall relationship when creatinine was greater than 133 μ mol/L; The protective effect of uric acid was stronger in smokers (OR=0.69, 95%CI: 0.61 – 0.78, P for interaction =0.003) and people with moderate physical activity (OR=0.70, 95%CI: 0.61 – 0.81, P for interaction =0.001). In addition, in the family monthly population level category, due to the small sample size of the subgroup who refused to answer and did not know the answer, this bias may lead to a slight interaction in the calculated results (P for interaction =0.049). Similarly, borderline in the diabetes subgroup a similar situation existed in the group.

Other subgroups showed a consistent protective trend. No significant interaction was found in general demographic characteristics such as age, sex, race, BMI subgroups, and common concomitant disease subgroups (P for interaction >0.05).

## Discussion

This cross-sectional study, which collected a nationally representative sample from 2005 to 2020, showed that after adjusting for confounders, there was a significant relationship between serum uric acid level and depression, and individuals with lower serum uric acid had a higher prevalence of depression than individuals with higher uric acid levels. Stratified analysis showed that the results were robust in the vast majority of the population. The dose-response relationship showed that uric acid was a risk factor for depression when uric acid was lower than 315.20 μmol/L, which was statistically significant.

In previous studies, some researchers have conducted relevant discussions, such as a study of middle-aged and elderly people from China mentioned in the introduction (24), while the population of this study is representative of participants from the United States, and the age is not limited to the elderly but still obtained similar results, which also shows the robustness of the results in different populations (24). In addition, clinical studies have shown that the uric acid level of patients with depression is significantly lower than that of patients with other cognitive impairment, and when these patients received standard antidepressant treatment for 5 weeks, the uric acid level returned to normal (32), which reflects the significance of the relationship between uric acid and depression from another side. In addition, depression can be subdivided into unipolar depression and bipolar depression. Some studies have compared the clinical characteristics of the two. This study proved that compared with patients with unipolar depression, patients with bipolar depression have higher serum uric acid levels and lower triglyceride levels (33). This may become a key biomarker to distinguish unipolar depression from bipolar depression.

In this study, there are some interesting phenomena worthy of attention. The most noticeable one is the reversal phenomenon in the low creatinine subgroup in **Figure 3**. High uric acid plays a protective role in depression in other groups, but it is a risk factor in this group alone. We speculate that creatinine, as a non enzymatic product of muscle phosphocreatine, its blood concentration mainly depends on the total muscle volume and glomerular filtration capacity (34, 35). It is a dual biomarker of muscle metabolism and renal function. Muscle tissue is the main site of glutathione synthesis (36). When muscle mass is significantly reduced, the systemic antioxidant capacity decreases (36, 37). In addition, low creatinine is often accompanied by glomerular hyperfiltration, resulting in unstable blood uric acid levels. This fluctuation may activate the inflammasome (such as NLRP3) (14, 38), which may lead to the conversion of uric acid into a depression risk factor due to the collapse of the antioxidant system.

Another interesting point is that the effect of uric acid on depression is more significant in the subgroup with moderate work activity, probably because there is an association between exercise intensity and depression, and exercise intensity is a strong predictor of depression (39). In addition, according to the symptom perception hypothesis, depression will play different roles in concurrent and retrospective physical reports, which will exaggerate retrospective physical reports (40). From the perspective of demography, the people who have moderate exercise as a healthier population have fewer comorbidities with other basic diseases (41), and the influence of uric acid on depression is less interfered with by other factors, so the result is more significant.

Another phenomenon is that uric acid is more closely related to depression in smokers, which may be related to the impact of tobacco exposure on the dopamine system in the body (42). Nicotine and tobacco particulate matter will affect the long-term imbalance of dopamine transport (43), which will lead to the generation of negative emotions and the excessive activation of microglia (44–46). Moreover, smokers themselves are exposed to more stress, and there are more stress reactions (47), which synergize with the functions of uric acid to promote inflammation and oxidative response, which together lead to this result (10).

We found that in patients with impaired glucose tolerance, higher uric acid was a risk factor for depression, but the opposite was true in patients with diabetes and normoglycemia. Our analysis may be that these correlations stem from complex two-way interactions, which involve many factors, including disorders of the nervous system and neuroendocrine system, structural changes of the hippocampus, inflammation, oxidative stress, and obesity (48, 49). In this study, the number of people with abnormal glucose tolerance is relatively small, and there may also be related bias. In short, there is no satisfactory mechanism to explain this result, which still needs further systematic mining.

Finally, compared with other studies, this study retained the participants who answered rejected in the family monthly prevalence level category because the relationship between financial status and depression was gradually explored (50, 51). Previous studies often only studied the exact data and excluded participants who refused to answer for various reasons. However, the avoidant response indicated that participants themselves were more likely to have risk factors or diseases (52, 53), which were closely related to depression (54), and they might need more social attention. Therefore, this study retained and analyzed this part of the participants. Although the sample size was not large enough and the confidence interval was wider than other groups, similar conclusions could still be drawn, indicating that uric acid and the relationship between depression remained robust in this group.

The mechanisms that can explain our study are different, and the role of uric acid itself is also diverse. In this study, we found that serum uric acid has a protective effect on depression in the vast majority of people. Low serum uric acid can significantly increase the risk of depression, probably because of the antioxidant effect of uric acid. As a highly effective free radical scavenger, uric acid can remove o2- and block the formation of peroxynitrite (ONOO^−^), playing a neuroprotective role (16, 55), but also improving calcium homeostasis, protecting cell membranes from damage and protecting mitochondria (56). In addition, uric acid protects the integrity of the blood-brain barrier, reduces the permeability of inflammatory cells, and has anti-inflammatory effects (19). From another perspective, uric acid, as a product of purine metabolism, is largely produced by food (8). Too low uric acid may mean food shortage, which is a blow both physically and psychologically, leading to a higher risk of depression (50, 51). However, we should be vigilant that although uric acid plays a protective role in most people, it can still play other roles. For example, when the creatinine level is low, the systemic antioxidant capacity decreases, the inflammasome is activated, and uric acid changes from “antioxidant guard” to “inflammatory trigger” (14, 36–38).

Although some studies of the same type have appeared in recent years (24, 25, 57), this study still has its advantages. We integrated adult data from eight NHANES cycles from 2005 to 2020, covering different races, ages, and socio-economic groups in the United States, with a sample size larger than previous similar studies. In addition, the confounding factors we focus on are more comprehensive (including demography, lifestyle, comorbidities, drugs, dietary nutrients, etc.), which is better than the adjustment dimensions of other studies. We also paid special attention to the special Responder groups that may be excluded by other studies and showed more care for the vulnerable groups.

There are still some limitations in this study. First, our study is a retrospective cross-sectional study and cannot show that there is a causal relationship between uric acid and depression. In the future, if there is an opportunity, longitudinal studies can be designed to explore this relationship. Secondly, although we included all potential confounding factors we could collect (including demography, comorbidities, diet, and common drugs) in the study, there are still some omissions. For example, we did not consider the impact of antidepressant drugs on this study, which needs to be supplemented by subsequent studies. Although our results are applicable to the vast majority of the population, the reversal results of the low creatinine population still need more experimental verification, and the use of glomerular filtration rate for the evaluation of renal function may get more accurate results. In addition, the participants in this study are all adults. The relationship between uric acid and depression in children needs further exploration.

## Conclusion

In the context of depression becoming a public health problem of great concern, this study used a nationally representative sample of American adults to explore the relationship between depression and uric acid. Our study showed that lower serum uric acid was associated with a higher prevalence of depression in American adults. For adults with low serum uric acid, attention should be paid to the prevention and treatment of depression. Further clinical and experimental studies are needed to verify their potential causality.

## Data Availability

Publicly available datasets were analyzed in this study. This data can be found here: https://www.cdc.gov/nchs/nhanes/index.htm.

## Supplementary material

Supplementary Figure 1 The logistic risk regression model of uric acid and depression, the adjusted hazard ratio (95% CI) of uric acid and depression is represented by a red curve. The cubic spline curve of the model has four nodes. Adjusted confounders included: Age, Gender, BMI, Race, Education level, Marital status, Family monthly poverty level category, Moderate work activity, Sleeping trouble, Smoking, Drinking, Total energy intake, High blood pressure, High cholesterol level, Diabetes,Protein (gm), Carbohydrate (gm), Total sugars (gm), Dietary fiber (gm), Total fat (gm), Total saturated fatty acids (gm), Total monounsaturated fatty acids (gm), Total polyunsaturated fatty acids (gm), Cholesterol (mg), Vitamin E as alpha-tocopherol (mg), Added alpha-tocopherol (Vitamin E) (mg), Retinol (mcg), Vitamin A, RAE (mcg), Alpha-carotene (mcg), Beta-carotene (mcg), Beta-cryptoxanthin (mcg), Lycopene (mcg), Lutein + zeaxanthin (mcg), Thiamin (Vitamin B1) (mg), Riboflavin (Vitamin B2) (mg), Niacin (mg), Vitamin B6 (mg), Total folate (mcg), Folic acid (mcg), Food folate (mcg), Folate, DFE (mcg), Total choline (mg), Vitamin B12 (mcg), Added vitamin B12 (mcg), Vitamin C (mg), Vitamin K (mcg), Calcium (mg), Phosphorus (mg), Magnesium (mg), Iron (mg), Zinc (mg), Copper (mg), Sodium (mg), Potassium (mg), Selenium (mcg), Caffeine (mg), Theobromine (mg),Hypertensive drugs,Hyperlipidemic drugs,Antidiabetics.
