# Supplementary material for: Relationship between uric acid and depression in American adults: findings from NHANES, 2005-2020
Source: Front Psychiatry. 2025 Jun 20;16:1544266. doi: 10.3389/fpsyt.2025.1544266 (PMC12226466; doi:10.3389/fpsyt.2025.1544266)
Supplement: Supplementary Figure 1 — The logistic risk regression model of uric acid and depression, the adjusted hazard ratio (95% CI) of uric acid and depression is represented by a red curve. The cubic spline curve of the model has four nodes. Adjusted confounders included: Age, Gender, BMI, Race, Education level, Marital status, Family monthly poverty level category, Moderate work activity, Sleeping trouble, Smoking, Drinking, Total energy intake, High blood pressure, High cholesterol level, Diabetes,Protein (gm), Carbohydrate (gm), Total sugars (gm), Dietary fiber (gm), Total fat (gm), Total saturated fatty acids (gm), Total monounsaturated fatty acids (gm), Total polyunsaturated fatty acids (gm), Cholesterol (mg), Vitamin E as alpha-tocopherol (mg), Added alpha-tocopherol (Vitamin E) (mg), Retinol (mcg), Vitamin A, RAE (mcg), Alpha-carotene (mcg), Beta-carotene (mcg), Beta-cryptoxanthin (mcg), Lycopene (mcg), Lutein + zeaxanthin (mcg), Thiamin (Vitamin B1) (mg), Riboflavin (Vitamin B2) (mg), Niacin (mg), Vitamin B6 (mg), Total folate (mcg), Folic acid (mcg), Food folate (mcg), Folate, DFE (mcg), Total choline (mg), Vitamin B12 (mcg), Added vitamin B12 (mcg), Vitamin C (mg), Vitamin K (mcg), Calcium (mg), Phosphorus (mg), Magnesium (mg), Iron (mg), Zinc (mg), Copper (mg), Sodium (mg), Potassium (mg), Selenium (mcg), Caffeine (mg), Theobromine (mg),Hypertensive drugs,Hyperlipidemic drugs,Antidiabetics. [file Table1.docx]

**Supplementary Table 1** Diet and medication characteristics of the study population

| **Characteristics** | **Total (n = 7507)** | **Normal (n = 6612)** | **depression (n = 895)** | **P** |
| --- | --- | --- | --- | --- |
|  |  |  |  |  |
| Protein (gm) | 75.90 ± 38.62 | 76.63 ± 38.57 | 70.50 ± 38.61 | <0.001 |
| Carbohydrate (gm) | 232.16 ± 112.16 | 232.18 ± 110.30 | 231.98 ± 125.09 | 0.964 |
| Total sugars (gm) | 100.81 ± 69.19 | 99.83 ± 66.66 | 108.03 ± 85.27 | 0.006 |
| Dietary fiber (gm) | 16.13 ± 9.79 | 16.37 ± 9.84 | 14.37 ± 9.23 | <0.001 |
| Total fat (gm) | 76.01 ± 43.33 | 76.51 ± 43.07 | 72.27 ± 45.10 | 0.006 |
| Total saturated fatty acids (gm) | 24.33 ± 15.20 | 24.43 ± 15.13 | 23.60 ± 15.70 | 0.128 |
| Total monounsaturated fatty acids (gm) | 27.25 ± 16.36 | 27.46 ± 16.27 | 25.74 ± 16.96 | 0.003 |
| Total polyunsaturated fatty acids (gm) | 17.65 ± 11.92 | 17.82 ± 11.89 | 16.38 ± 12.07 | <0.001 |
| Cholesterol (mg) | 288.10 ± 232.52 | 291.00 ± 233.71 | 266.65 ± 222.55 | 0.003 |
| Vitamin E as alpha-tocopherol (mg) | 7.84 ± 5.94 | 7.92 ± 5.87 | 7.25 ± 6.40 | 00.001 |
| Added alpha-tocopherol (Vitamin E) (mg) | 0.59 ± 3.02 | 0.58 ± 2.97 | 0.60 ± 3.32 | 0.917 |
| Retinol (mcg) | 407.21 ± 530.94 | 409.03 ± 507.88 | 393.80 ± 677.59 | 0.421 |
| Vitamin A, RAE (mcg) | 609.30 ± 654.26 | 617.26 ± 640.03 | 550.50 ± 748.82 | 0.004 |
| Alpha-carotene (mcg) | 378.84 ± 1025.42 | 394.46 ± 1062.03 | 263.45 ± 687.38 | <0.001 |
| Beta-carotene (mcg) | 2189.78 ± 4054.91 | 2255.00 ± 4098.56 | 1707.94 ± 3683.08 | <0.001 |
| Beta-cryptoxanthin (mcg) | 101.21 ± 309.89 | 102.95 ± 307.67 | 88.42 ± 325.73 | 0.188 |
| Lycopene (mcg) | 4650.55 ± 8979.53 | 4691.22 ± 8656.94 | 4350.11 ± 11076.61 | 0.286 |
| Lutein + zeaxanthin (mcg) | 1555.89 ± 3539.98 | 1585.93 ± 3608.85 | 1334.02 ± 2974.73 | 0.046 |
| Thiamin (Vitamin B1) (mg) | 1.50 ± 0.85 | 1.52 ± 0.85 | 1.41 ± 0.83 | <0.001 |
| Riboflavin (Vitamin B2) (mg) | 1.97 ± 1.15 | 1.97 ± 1.12 | 1.92 ± 1.38 | 0.231 |
| Niacin (mg) | 23.28 ± 13.78 | 23.44 ± 13.58 | 22.04 ± 15.09 | 0.004 |
| Vitamin B6 (mg) | 1.91 ± 1.49 | 1.93 ± 1.42 | 1.77 ± 1.92 | 0.003 |
| Total folate (mcg) | 370.53 ± 225.42 | 374.42 ± 226.21 | 341.80 ± 217.42 | <0.001 |
| Folic acid (mcg) | 163.44 ± 162.07 | 164.75 ± 162.50 | 153.73 ± 158.61 | 0.056 |
| Food folate (mcg) | 207.10 ± 132.79 | 209.67 ± 133.31 | 188.07 ± 127.38 | <0.001 |
| Folate, DFE (mcg) | 484.85 ± 324.48 | 489.66 ± 325.52 | 449.33 ± 314.59 | <0.001 |
| Total choline (mg) | 321.19 ± 187.63 | 324.66 ± 188.11 | 295.53 ± 182.15 | <0.001 |
| Vitamin B12 (mcg) | 4.76 ± 5.89 | 4.79 ± 5.70 | 4.58 ± 7.18 | 0.311 |
| Added vitamin B12 (mcg) | 0.85 ± 2.38 | 0.85 ± 2.33 | 0.83 ± 2.70 | 0.820 |
| Vitamin C (mg) | 80.82 ± 87.40 | 81.68 ± 85.28 | 74.45 ± 101.51 | 0.020 |
| Vitamin K (mcg) | 112.25 ± 179.44 | 113.89 ± 181.81 | 100.16 ± 160.43 | 0.032 |
| Calcium (mg) | 863.13 ± 531.36 | 865.93 ± 530.67 | 842.44 ± 536.28 | 0.215 |
| Phosphorus (mg) | 1259.99 ± 618.11 | 1269.08 ± 618.26 | 1192.86 ± 613.18 | <0.001 |
| Magnesium (mg) | 280.77 ± 137.96 | 283.32 ± 138.06 | 261.94 ± 135.87 | <0.001 |
| Iron (mg) | 14.03 ± 8.22 | 14.19 ± 8.29 | 12.88 ± 7.63 | <0.001 |
| Zinc (mg) | 10.56 ± 7.11 | 10.67 ± 7.22 | 9.72 ± 6.16 | <0.001 |
| Copper (mg) | 1.21 ± 0.95 | 1.22 ± 0.92 | 1.14 ± 1.16 | 0.024 |
| Sodium (mg) | 3264.98 ± 1683.31 | 3295.03 ± 1678.68 | 3042.98 ± 1701.69 | <0.001 |
| Potassium (mg) | 2533.63 ± 1189.51 | 2562.94 ± 1189.70 | 2317.15 ± 1166.16 | <0.001 |
| Selenium (mcg) | 105.95 ± 59.70 | 106.92 ± 59.62 | 98.82 ± 59.90 | <0.001 |
| Caffeine (mg) | 153.94 ± 198.87 | 150.82 ± 183.44 | 177.02 ± 287.42 | 0.008 |
| Theobromine (mg) | 32.09 ± 70.46 | 31.50 ± 65.97 | 36.44 ± 97.33 | 0.141 |
| Hypertensive drugs, n(%) |  |  |  | 0.908 |
| Yes | 6827 (90.94) | 6014 (90.96) | 813 (90.84) |  |
| No | 680 (9.06) | 598 (9.04) | 82 (9.16) |  |
| Hyperlipidemic drugs, n(%) |  |  |  | 0.758 |
| Yes | 4847 (64.57) | 4265 (64.50) | 582 (65.03) |  |
| No | 2660 (35.43) | 2347 (35.50) | 313 (34.97) |  |
| Antidiabetics, n(%) |  |  |  | <0.001 |
| Yes | 760 (10.12) | 618 (9.35) | 142 (15.87) |  |
| No | 6747 (89.88) | 5994 (90.65) | 753 (84.13) |  |

Continuous variables are expressed as mean (SD) for normally distributed data and as median with interquartile range for non normally distributed data. Categorical variables are expressed as percentages.
