# Supplementary material for: Relationship between uric acid and depression in American adults: findings from NHANES, 2005-2020
Source: Front Psychiatry. 2025 Jun 20;16:1544266. doi: 10.3389/fpsyt.2025.1544266 (PMC12226466; doi:10.3389/fpsyt.2025.1544266)
Supplement: Supplementary file 2 [file Table2.docx]

**Supplementary Table 2** The relationship between uric acid and the risk of depression

| **Uric acid,μmol/L** | **Model1** | |  | **Model2** | |
| --- | --- | --- | --- | --- | --- |
|  | **OR (95%CI)** | ***P*** |  | **OR (95%CI)** | ***P*** |
| Tertiles |  |  |  |  |  |
| T1 (≤279.6) | 1.00 (Reference) |  |  | 1.00 (Reference) |  |
| T2 (279.6-350.9) | 0.73 (0.61 ~ 0.88) | <0.001 |  | 0.81 (0.66 ~ 0.98) | 0.034 |
| T3 (≥350.9) | 0.70 (0.59 ~ 0.83) | <0.001 |  | 0.81 (0.66 ~ 0.98) | 0.033 |
| OR: Odds Ratio, CI: Confidence Interval | | | | | |
| Model1: Crude | | | | | |
| Model2: Adjust: Age, Gender, BMI, Race, Education level, Marital status, Family monthly poverty level category, Moderate work activity, Sleeping trouble, Smoking, Drinking, Total energy intake, High blood pressure, High cholesterol level, Diabetes,Protein (gm), Carbohydrate (gm), Total sugars (gm), Dietary fiber (gm), Total fat (gm), Total saturated fatty acids (gm), Total monounsaturated fatty acids (gm), Total polyunsaturated fatty acids (gm), Cholesterol (mg), Vitamin E as alpha-tocopherol (mg), Added alpha-tocopherol (Vitamin E) (mg), Retinol (mcg), Vitamin A, RAE (mcg), Alpha-carotene (mcg), Beta-carotene (mcg), Beta-cryptoxanthin (mcg), Lycopene (mcg), Lutein + zeaxanthin (mcg), Thiamin (Vitamin B1) (mg), Riboflavin (Vitamin B2) (mg), Niacin (mg), Vitamin B6 (mg), Total folate (mcg), Folic acid (mcg), Food folate (mcg), Folate, DFE (mcg), Total choline (mg), Vitamin B12 (mcg), Added vitamin B12 (mcg), Vitamin C (mg), Vitamin K (mcg), Calcium (mg), Phosphorus (mg), Magnesium (mg), Iron (mg), Zinc (mg), Copper (mg), Sodium (mg), Potassium (mg), Selenium (mcg), Caffeine (mg), Theobromine (mg),Hypertensive drugs,Hyperlipidemic drugs,Antidiabetics. | | | | | |
|  | | | | | |
