# Supplementary figures and images for: Relationship between uric acid and depression in American adults: findings from NHANES, 2005-2020
Source: Front Psychiatry. 2025 Jun 20;16:1544266. doi: 10.3389/fpsyt.2025.1544266 (PMC12226466; doi:10.3389/fpsyt.2025.1544266)

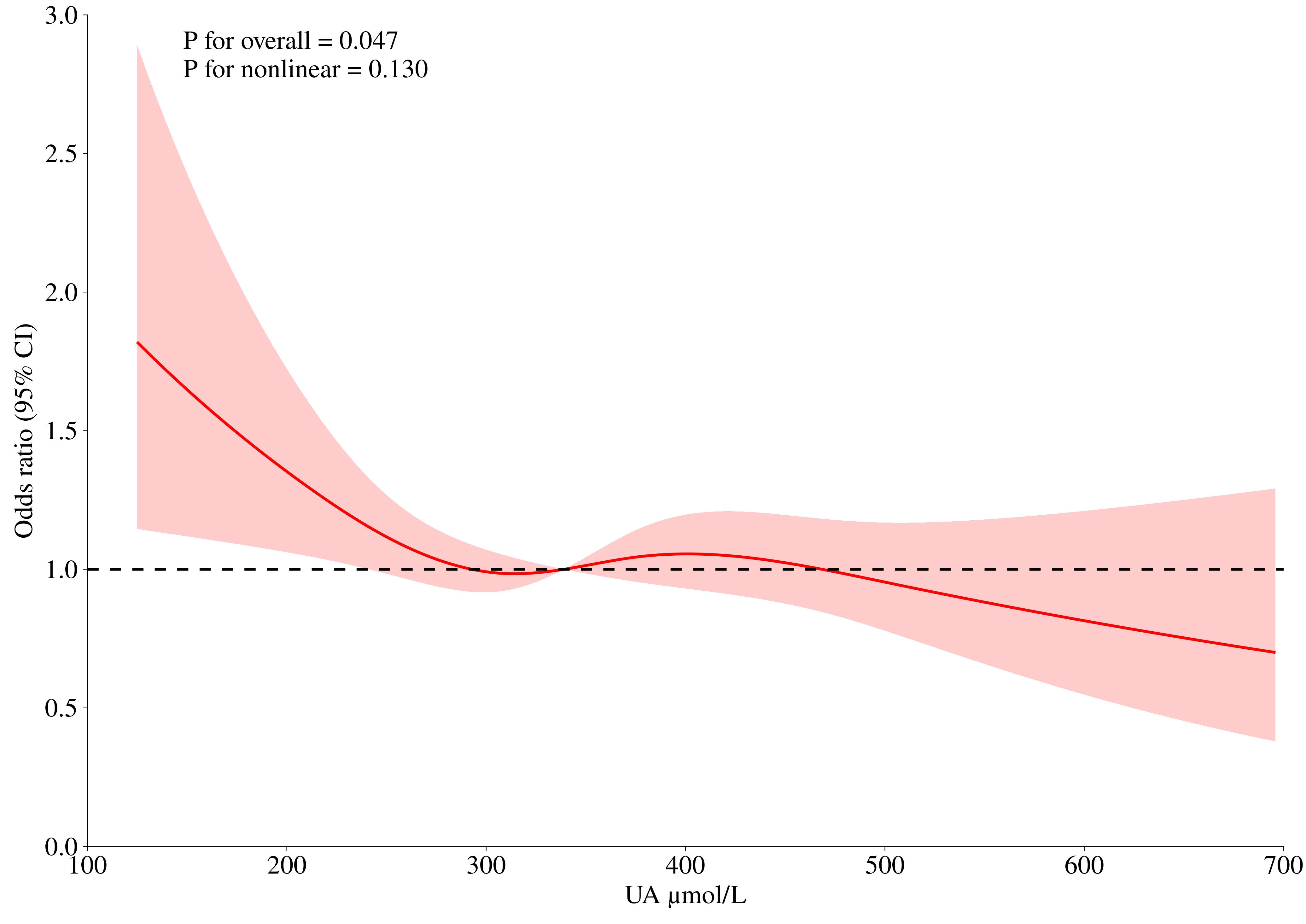

Supplement: Supplementary file 3 [file Image1.tiff]
